# Microstructure and performance of laser cladding 17-4PH-xTiC coatings on harvester blades

**DOI:** 10.1371/journal.pone.0336947

**Published:** 2025-11-20

**Authors:** Dian Yu Luo, Chunjing Liu, Tianlu Wei, Chao Wang, Hairui Ma, Shichun Jiang

**Affiliations:** 1 College of Mechanical and Vehicle Engineering, Bengbu University, Bengbu, China; 2 College of Intelligent Manufacturing, Anhui Science and Technology University, Chuzhou, China; 3 Anhui Province Additive Manufacturing Engineering Research Center, Bengbu, China; 4 School of Ruian, Wenzhou Polytechnic, Wenzhou, China; 5 Anhui Yucheng Laser Technology Co. Ltd., Bengbu, China; University of Vigo, SPAIN

## Abstract

To address the need for extending the service life of combine harvester blades, this study employs laser cladding technology to prepare 17–4PH-xTiC coatings with varying TiC content on 65Mn steel substrates. The effect of TiC content on the microstructure and properties of the coatings was investigated. Specifically, the microstructure of the coatings was observed by scanning electron microscopy and optical microscopy. X-ray diffraction was used to analyze the phase composition, a Vickers hardness tester measured the hardness, and a friction and wear testing machine evaluated the wear resistance. The coating composition with the optimal performance was selected to prepare reinforced coatings on the surface of harvester blades, followed by field tests. Results indicated that when the TiC content in the coating reached 40%, cracks appeared on the surface, and the number of cracks gradually decreased as laser cladding progressed. The cross-sectional microstructure of the coatings mainly featured columnar crystals, columnar dendrites, cell crystals, and unmelted TiC particles. The proportion of secondary precipitated fishbone-like TiC particles also increased, refining the microstructure. The coatings primarily consisted of α-Fe, TiC, γ-Fe, and Cr_23_C_6_. The average hardness of the 17–4PH-xTiC coatings ranged from 359 HV_0.2_ to 556 HV_0.2_. Under room temperature conditions, the primary wear mechanisms of the coatings were oxidative wear and adhesive wear. The wear loss of the 10–40% TiC coatings was reduced by 44.1% to 68.1%, respectively, compared to the substrate (65Mn). Field tests showed that the laser-cladded blades maintained a relatively sharp edge even after harvesting wheat for 120 h, whereas the commercially available heat-treated blades had completely failed. Compared to the commercial blade, the wear of the laser-cladded blade was decreased by 67%. This study successfully applied wear-resistant laser cladding coatings on the surface of harvester blades with small substrate thickness, significantly extending their service life.

## Introduction

Operating in harsh conditions, agricultural harvester blades need to endure contact with soil, sand, and other hard substances, which causes wear and failure. The blades are vital components in the harvesting process of agricultural harvester, and their service life directly affects the machinery’s operational efficiency. Therefore, implementing surface modification to extend the service life of agricultural blades is essential for enhancing the efficiency and economic benefits of agricultural harvester [1–3]. The commonly used agricultural harvester blade material, 65Mn, is typically subjected to heat treatment processes such as quenching and tempering to enhance its wear resistance and toughness [4]. However, solely relying on heat treatment for blade reinforcement offers limited performance improvements and is inadequate for meeting the wear resistance demands of agricultural harvester blades under harsh working conditions [5]. For further improving wear resistance, surface treatment techniques are often employed to create wear-resistant coatings on the substrate surface [6]. Common surface treatment methods include surfacing, thermal spraying, magnetron sputtering, and laser cladding [7].

Currently, numerous researchers are intensively studying the preparation of wear-resistant coatings on agricultural harvester blade surfaces to enhance their durability. Khan et al. [8] employed high-speed pure oxygen combustion technology to develop coatings with varying Cr content on the surface of EN8 steel to enhance the wear resistance of the substrate. The study revealed that the addition of chromium to the coating led to the formation of new carbides (C_2_Cr_3_ and Cr_7_C_3_). Nevertheless, field tests were not conducted, and the effectiveness of this technology in practical engineering applications remains unverified. Bazhin et al. [9] applied surfacing welding to develop wear-resistant coatings on the surfaces of plow bodies. Practical verification demonstrated that the lifespan of reinforced plow bodies was 2.5-3 times longer than that of untreated ones. Nonetheless, surfacing welding considerably affects the substrate and is only suitable for strengthening the surfaces of agricultural harvester blades with thick substrates. For thin-walled agricultural harvester blades, the surface treatment process can cause the edges to melt, preventing effective reinforcement of blades with thin edges. Dilay [10] utilized atmospheric plasma spray to prepare Ni-WC coatings on cultivator blade surfaces. The results showed good adhesion between the coatings and the blade, with the hardness of the reinforced coatings increasing by 1.5 times and the wear resistance by 3 times compared with the substrate. However, the field test soil was sandy loam, and the increased lifespan in hilly terrain fields remains unproven.

Unlike traditional surface treatment techniques, laser cladding involves melting both the substrate and the cladding material using a laser beam, followed by the solidification of the melted material to form a new cladding layer. It offers advantages such as strong bonding strength, minimal effect on the substrate properties, and a wide selection range of cladding materials [11–13]. Particularly for thin agricultural harvester blades, laser cladding is beneficial given its low heat input, which protects the substrate against melting and damage during the coating process [14]. Xu et al. [15] employed laser cladding technology to fabricate AlCoCrFeNi-WC coatings on the surface of 65Mn silage blades and investigated the coating’s effect on blade wear-resistant performance. Field tests demonstrated that the wear-resistant coating produced by single-sided laser cladding exhibited a self-sharpening effect, effectively extending the service life of the blades. Despite these advantages the high cost of the cladding powder has hindered large-scale production. Chen et al. [16] prepared Fe-xMo coatings on a 65Mn substrate. The results showed that as the Mo content increased, the coating’s microstructure became more refined, and its impact resistance improved slightly. On the other hand, an excessive amount of molybdenum increased the likelihood of cracking in the coating, which adversely affected its overall strength. Shi et al. [17] employed laser cladding technology to apply a Ni60A/SiC coating on the surface of blades, aiming to address wear-induced failure in forage mixer blades. The study demonstrated that the addition of La_2_O_3_ significantly enhanced the wear resistance of the coating. Compared to commercial coatings, the wear depth of the Ni60A/SiC coating with 2% La_2_O_3_ was reduced by 86%. However, as the results were solely based on theoretical analysis without experimental validation, the reliability of the findings remains uncertain.

The application of wear-resistant coatings on agricultural harvester blade surfaces can effectively enhance their abrasion resistance and prolong service life. When utilizing laser cladding technology for blade reinforcement, the selection of appropriate powder systems constitutes a critical practical consideration to meet engineering application requirements. The incorporation of ceramic reinforcement phases into metallic coatings demonstrates beneficial effects in improving wear resistance properties. However, the strengthening strategy for agricultural cutting implements necessitates comprehensive consideration of both wear resistance and fracture toughness. Excessive coating hardness may induce impact-induced failure during crop harvesting operations due to collision stresses, thus requiring balanced mechanical property optimization [18–20]. As a precipitation-hardened martensitic stainless steel, 17−4PH has excellent corrosion resistance and outstanding tensile and impact strength, making it widely used in aviation, aerospace, and laser repair fields. However, 17−4PH suffers from low hardness and poor wear resistance [21–23]. Current scholarly attention to the fabrication of 17–4PH-TiC composite coatings via laser cladding technology remains limited, particularly in the context of agricultural machinery component reinforcement. The metallurgical compatibility between 17−4PH stainless steel matrix and TiC ceramic particles, along with the interfacial bonding characteristics under high-energy laser processing conditions, has not been systematically investigated. Thus, this study investigates laser cladding of 17−4PH + xTiC coatings with different contents on 65Mn substrates, focusing on macrocracks, microstructure, phase composition, and wear resistance. The best powder ratio is chosen to prepare coatings on harvester blades, with field trials conducted to confirm the reinforcement effect. The prepared coatings exhibit excellent mechanical properties, and the wear resistance is greatly improved compared with that of heat-treated 65Mn blades. This study provides a reference for the surface strengthening process of thin-walled components and their material selection.

## Materials and methods

### Materials

**Fig 1:**
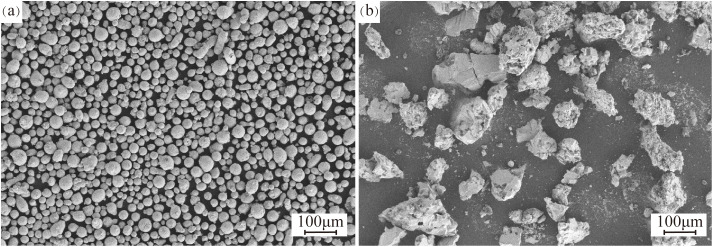
SEM morphology of power: (a)17−4PH; (b)TiC.

The substrate selected for the laser cladding experiment was consistent with the blade material (i.e., 65Mn). Prior to the experiment, the surface of the 65Mn substrate was cleaned using a sandblasting machine to remove the oxide layer and grease, in order to prevent the cladding layer from delaminating. The cladding material was 17−4PH alloy powder mixed with different proportions of TiC powder, ball-milled for 3 h before the experiment. The surface morphology of the powder is shown in Fig 1. For experimental accuracy, the powder and the cleaned 65Mn substrate were placed in an oven and heated to 100°C, and maintained at that temperature for 2 h before cladding. This step prevented moisture in the powder from creating pores during cladding, which could affect the coating quality, and prevented the powder from clogging the feeding system.

### Coating preparation

**Fig 2:**
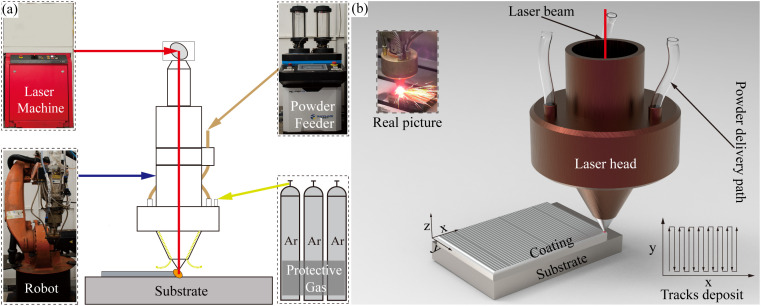
Schematic of experimental equipment and process. (a) Laser cladding system; (b) Laser cladding process.

This experiment utilized a 3 kW coaxial powder-feeding fiber laser cladding device (LCD3000DR), as depicted in Fig 2, with a spot diameter of 3 mm. The detailed information of the laser cladding equipment can be found in reference [24]. According to prior research, the laser cladding process parameters were as follows: laser power of 1600 W, scanning speed of 15 mm/s, powder feeding speed of 10.7 g/min, overlap rate of 50%, powder feeding gas flow rate of 6 L/min, and shielding gas flow rate of 10 L/min.

### Microstructural characterization and wear testing

Metallographic samples were cut along the vertical direction of the coating using a wire electrical discharge machine. Additionally, circular wear test samples with a diameter of 25 mm were prepared. The XRD test samples were consistent with the metallographic samples and the cut samples were polished. Metallographic samples were polished and etched with aqua regia for 5–10 s, after which the coating’s microstructure was examined using a Zeiss Axio Imager 2 optical microscope. The hardness of the coating cross-section was measured with an HV-1000Z Vickers hardness tester, with measurements taken from the coating surface to the substrate. To ensure data accuracy, un-melted TiC particles within the coating were avoided during the testing. A load of 200 g was applied for 10 s during the indentation process.

The friction and wear samples were initially polished using a surface grinder. Subsequently, dry sliding wear tests (CHMT-23 tribometer) were performed at room temperature, using a 6 mm diameter Si3N4 spherical ball under a normal load of 50 N, with a sliding speed of 500 rpm on the coated surface. After the tests, the wear mass loss was measured with a YT3024 precision electronic balance, featuring a sensitivity of 0.1 mg. Each sample was measured 3 times to minimize random errors. After measurement, the wear track’s three-dimensional morphology was examined using an Rtec UP-Sigma white light interferometer. Additionally, the wear track’s microstructure was analyzed using a Zeiss Gemini scanning electron microscope. Energy-dispersive X-ray spectroscopy (EDS) was used to analyze the elemental distribution in the coating and the wear track, providing insights into the wear mechanisms of the coating.

### Field test conditions and equipment

The test site, which is located in Quanjiao County, Chuzhou City, Anhui Province (N 32°10′56″, E 118°18′72″). The wheat harvested in this experiment has an average plant height of approximately 74 cm, with a stem moisture content of about 65%. After harvest, the remaining wheat stubble height is 15 cm. The experimental equipment is a Weichai Lovol RG70 combine harvester, with a cutting width of 2.2 m, a harvesting speed of 10 km/h, and a straw chopping length of about 20–40 mm.

## Results and discussions

### Macroscopic Morphology

**Fig 3:**
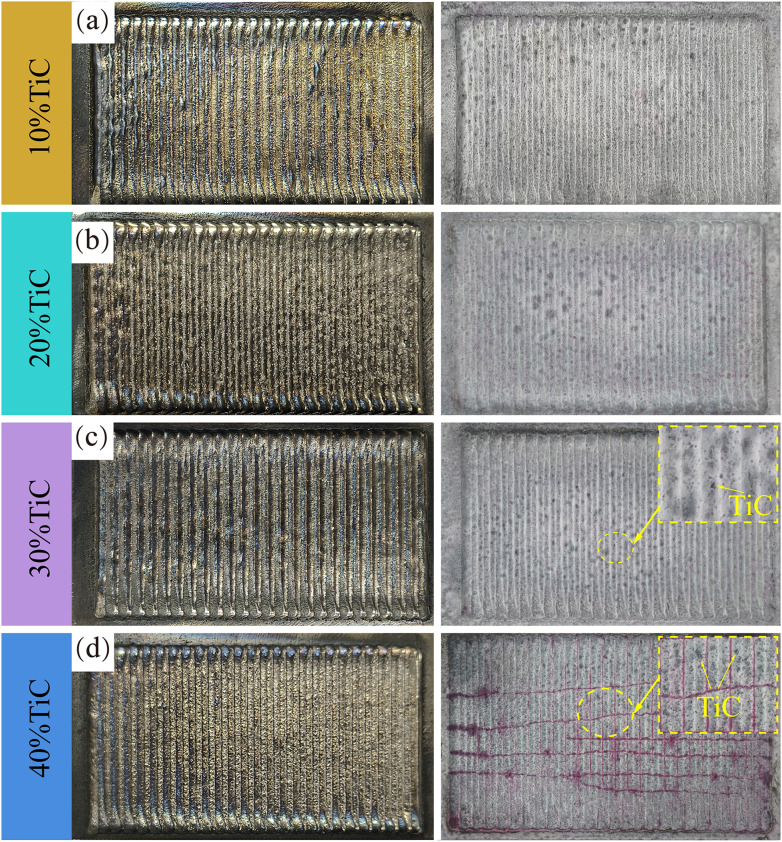
Macroscopic morphology of 17-4PH-xTiC coatings. (a) 10%TiC; (b) 20%TiC; (c) 30%TiC; (d) 40%TiC.

**Fig 4:**
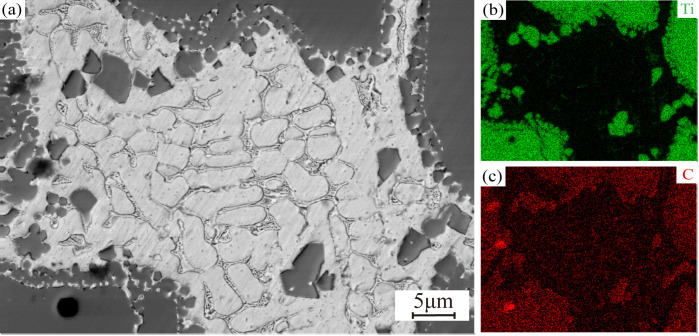
Unmolten particles in the coating. (a) the image of the particle; (b) and (c) the EDS analysis.

Unmolten particles are observed on the surfaces of all coatings, as demonstrated in Fig 3. Elemental analysis of these particles, presented in Fig 4, confirms their identification as unmolten TiC. The cause of this phenomenon can be attributed to the fact that TiC has a lower density than the 17−4PH alloy, which causes TiC particles to float to the surface of the molten pool, forming visible TiC particles on the coating surface. When the TiC content is between 10% and 30%, no significant cracks are observed on the coating surface. However, when the TiC content reaches 40%, a large number of spider-web-like cracks appear on the coating surface. These cracks are not only perpendicular to the cladding trajectory but are also observed to be parallel to the cladding trajectory during the final stage of the process. These cracks, representing a prevalent type of structural imperfection in laser-clad surfaces, have been identified as critical factors that compromise the cohesive continuity of the clad layer through their propagation mechanisms [25]. The crack formation process can be divided into two stages: during the early stages of cladding, small, densely packed cracks primarily form. As the cladding process progresses to about two-thirds completion, the cracks gradually transition from fine, dense cracks to larger ones that penetrate the entire coating. The main reason for this phenomenon is the high melting point of TiC, which prevents it from fully melting in the molten pool. Under the same energy input conditions, an increase in TiC content reduces the proportion of melted TiC, leading to an increase in the number of unmolten TiC particles. These unmolten TiC particles disrupt the continuity of the coating, resulting in the formation of cracks on the surface. As the cladding process enters the final third, the overall temperature of the substrate and coating increases due to the higher thermal input, which preheats the unmolten areas to some extent, alleviating the thermal stresses induced by the process. This improvement in crack formation is consistent with the findings in the study by [26].

### Microstructure analysis

**Fig 5:**
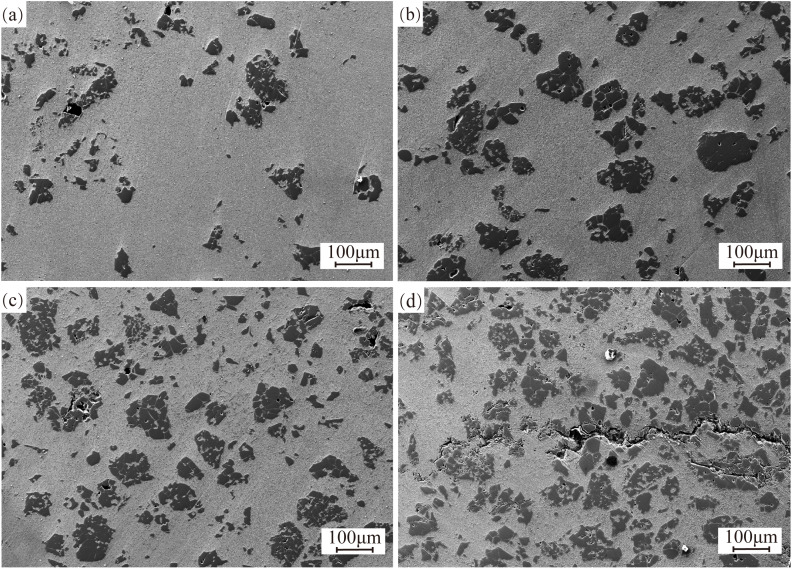
SEM morphology of 17-4PH-xTiC coatings. (a) 10%TiC; (b) 20%TiC; (c) 30%TiC; (d) 40%TiC.

The scanning electron microscopy (SEM) morphology of the coating cross-section at various TiC contents is shown in Fig. 5, with the samples sectioned perpendicular to the laser cladding track. As the TiC content increases, the proportion of unmelted TiC particles in the coating also rises and becomes more uniformly distributed. These unmelted TiC particles can improve the wear resistance of the coating. However, cracks and holes occur on the surface of the unmelted TiC particles, which may be due to defects such as porosity and cracks that emerged on the surface of the selected TiC powder during the preparation process (Fig. 1(b)). When the addition of TiC is increased to 40%, cracks appear in the coating, primarily located at the edges of the unmelted TiC particles. This phenomenon may be attributed to the large difference in the coefficient of thermal expansion between TiC and 17−4PH alloy, resulting in excessive internal stresses due to the inconsistent shrinkage rate during the cooling process, ultimately leading to the formation of cracks [27]. At the same time, no strong interfacial bonding or alloying structure is formed between the unmolten TiC particles and the coating matrix. This weak interfacial bonding can lead to delamination or crack formation at the interface when the coating is subjected to external forces or thermal cycling [28]. Additionally, cracks tend to propagate rapidly along the interface, reducing the coating’s plasticity and further exacerbating its susceptibility to cracking.

**Fig 6:**
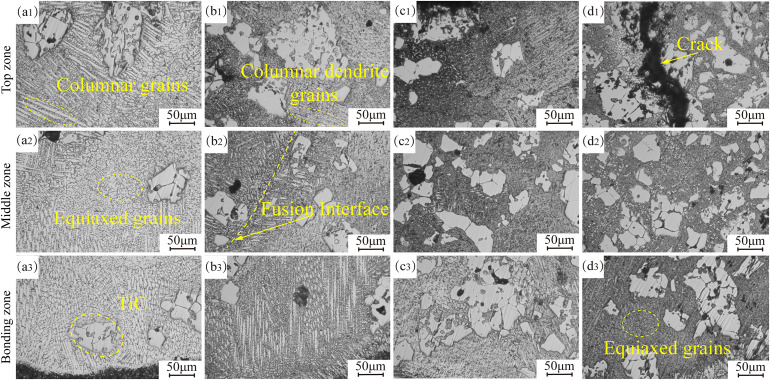
Metallographic organization of 17-4PH-xTiC coatings. (a) 10%TiC; (b) 20%TiC; (c) 30%TiC; (d) 40%TiC.

To elucidate the influence of TiC content variations on coating microstructures, further metallographic characterization was performed on the coating specimens (Fig 6). From Fig 6(a1) to (d1), the overall grain size of the coating exhibited a gradual refinement trend, with the most pronounced grain refinement observed in the top region of the coating where the grain size decreased from 145 μm to 10 μm. In coatings with 10%−30% TiC, the microstructure at the surface is primarily composed of columnar grains, with a small amount of dendrites. When the TiC content reaches 40%, cracks approximately 40 μm in width appear at the coating surface, primarily concentrated at the edges and centers of the unmolten TiC particles. Notably, as shown in Fig 6(b2), at the overlap region of the coating, the material experiences reheating due to the influence of the subsequent laser scan path, creating a heat-affected zone. The microstructure in this region undergoes a laser remelting-like effect due to the thermal input, resulting in coarsened recrystallization of the grains at the overlap. From Fig 6(a3) to (d3), it can be observed that the coating’s bottom layer mainly consists of columnar grains, columnar dendrites, and a small amount of equiaxed grains, with the columnar grains growing toward the top of the molten pool. This phenomenon occurs because, at the beginning of the cladding process, the substrate temperature is relatively low, and there is a significant temperature gradient between the molten coating and the substrate. When the molten pool comes into contact with the substrate, columnar grains grow in the direction opposite to the heat dissipation of the molten pool [29]. As the TiC content increases, the number of unmolten TiC particles at the coating’s bottom increases, while the columnar grains at the base of the molten pool gradually disappear. The grain size in the coating continues to refine, as some TiC particles decompose under the high-energy laser, forming new nucleation sites that suppress grain growth [30]. Furthermore, the large TiC particles at the bottom of the molten pool hinder grain growth [31], and this dense microstructure contributes to enhanced hardness and wear resistance of the coating.

**Fig 7:**
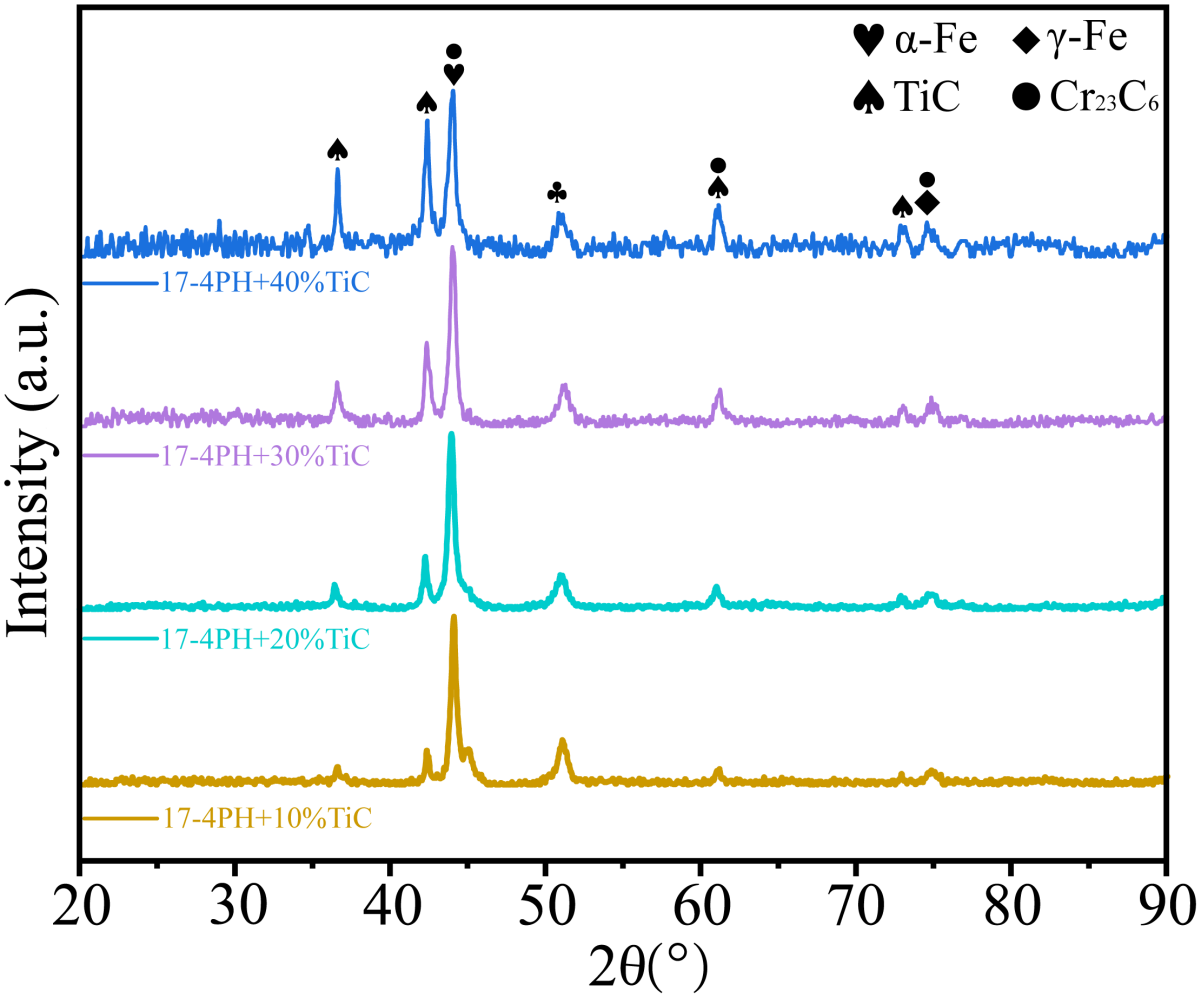
XRD patterns of 17-4PH-xTiC coatings.

The phase identification results obtained through XRD analysis, as depicted in Fig 7, demonstrate the compositional modifications induced by varying TiC additions. The primary constituents of the coatings are α-Fe, TiC, γ-Fe, and Cr_23_C_6_. Upon decomposition in the molten pool, TiC yields Ti and C elements. Subsequently, the liberated C element reacts with Cr to produce Cr_23_C_6_. The position of the TiC signal in the spectrum aligns with the findings in reference [32]. As the TiC content increases, a gradual increase in the peak intensity of TiC is observed. Since the laser cladding process parameters remain unchanged, this suggests that the amount of unmolten TiC in the coating also increases correspondingly.

**Fig 8:**
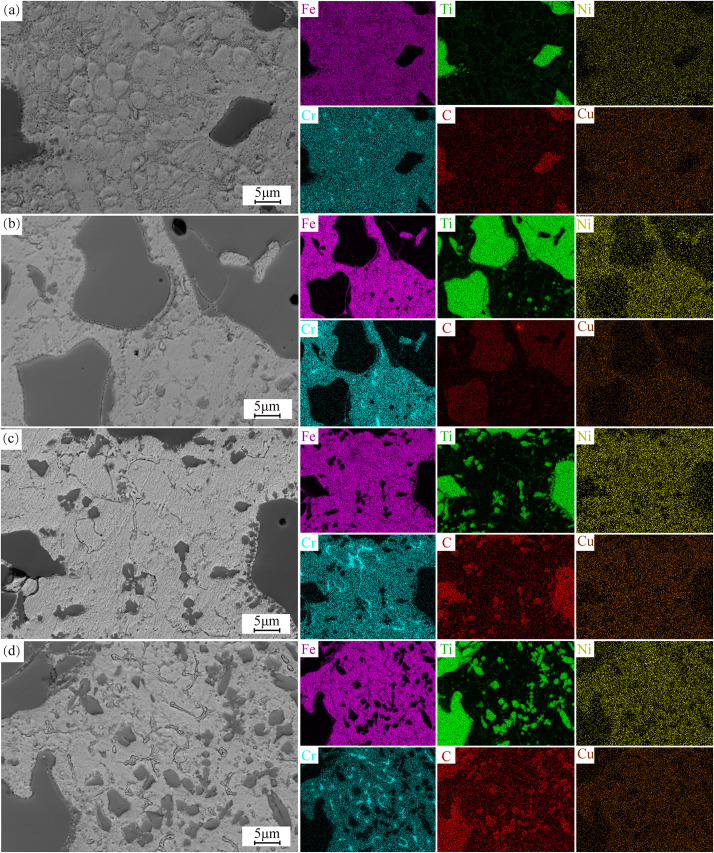
SEM morphology and EDS surface-scanning energy spectra of the cross-sectional organization of 17-4PH-xTiC coatings. (a) 10%TiC; (b) 20%TiC; (c) 30%TiC; (d) 40%TiC.

Fig 8 displays the SEM micrographs and energy-dispersive EDS elemental maps of coatings with graded TiC additions, highlighting the spatial distribution of constituent elements and microstructural evolution. The coating primarily consists of Fe, Ti, Cr, C, Ni, and Cu. As the TiC content increases, a greater number of smaller TiC particles decompose under the high-energy laser. The decomposed TiC exists in the molten pool as Ti and C elements [33]. During cooling, due to the high melting point of TiC, these Ti and C elements preferentially recombine to form fine, petal-like TiC particles, which are dispersed throughout the microstructure. Additionally, Ti signals are observed within the grains, where Ti contributes to solid-solution strengthening. At the grain boundaries, Ti-enriched zones are also observed, with C detected in these regions. This suggests that TiC decomposes and re-forms into finer TiC particles at the grain boundaries, contributing to the secondary phase strengthening of the coating. However, the precipitation of TiC at the grain boundaries increases the coating’s hardness at the expense of toughness. In all coatings, small TiC particles are observed at the edges of larger TiC particles, consistent with the findings in reference [34]. This confirms that some TiC particles decompose during the laser cladding process and that the TiC particles at the grain boundaries originate from decomposed TiC. Notably, Cr-enriched zones are also observed at the grain boundaries, overlapping significantly with the C element signal. This suggests that C reacts with Cr in the molten pool to form chromium carbides (Cr_23_C_6_). These hard carbide phases, dispersed along the grain boundaries, significantly enhance the coating’s hardness and wear resistance [35]. However, the presence of chromium carbides at the grain boundaries reduces the coating’s toughness, making it more brittle.

### Microhardness

**Fig 9:**
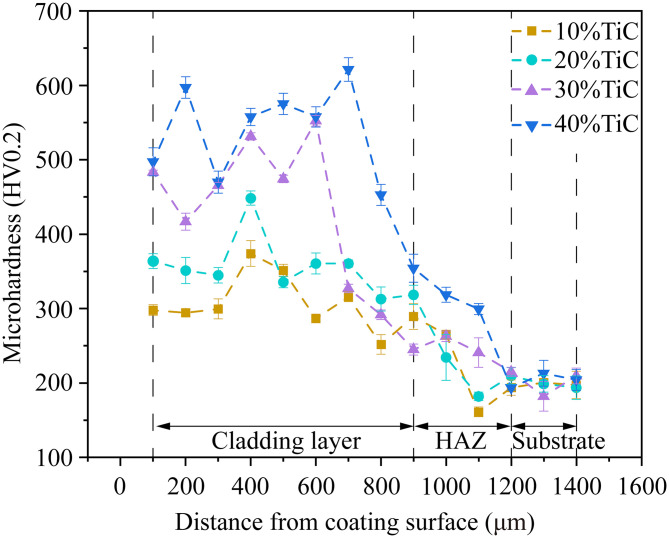
Hardness distribution of 17-4PH-xTiC coatings cross section.

The hardness distribution across the cross-section of 17−4PH + xTiC coatings was delineated in Fig 9, including the hardness values for the coating, heat-affected zone (HAZ), and substrate. The observed hardness trend indicates that the coating’s hardness is significantly higher than that of the substrate. Additionally, the hardness of the heat-affected zone is also higher than that of the substrate. This can be attributed to the rapid cooling effect at the substrate surface during the laser cladding process, which results in the formation of a martensitic microstructure in the HAZ, thereby increasing its hardness [36]. The hardness data reveals a linear relationship between the coating’s cross-sectional hardness and TiC content, with the average hardness of the coating increasing as the TiC content rises. The average surface hardness values for the 17–4PH-10%TiC, 20%TiC, 30%TiC, and 40%TiC coatings are 359, 402, 556, and 572 HV_0.2_, respectively, while the average hardness of the substrate is only 207 HV_0.2_. This can be attributed to the refining effect of added TiC on the coating’s microstructure. Additionally, the fine TiC particles that precipitate and disperse throughout the coating further enhance its hardness [37].

### Friction Coefficient and Wear Mechanism

**Fig 10:**
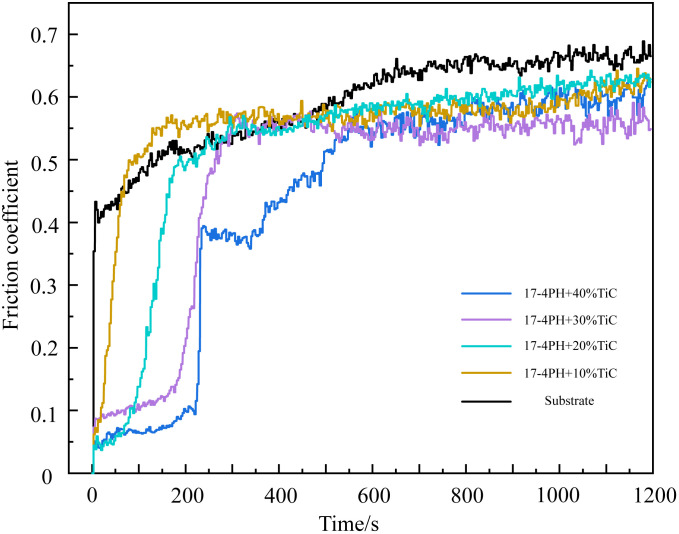
Friction coefficient curve of 17-4PH-xTiC coatings.

The relationship between the friction coefficient and time for coatings with varying TiC contents and the substrate is demonstrated in Fig 10 under room temperature conditions. The process can be divided into two stages: the initial running-in stage and the stable wear stage. During the running-in stage, the friction coefficients of the substrate, 10% TiC coating, and 20% TiC coating show a significant increase. Specifically, the friction coefficient of the substrate rapidly rises to around 0.44 within the first 0–10 s, while the running-in period for the 10% TiC and 20% TiC coatings extends to 0–50 s and 0–150 s, respectively. This behavior is attributed to point contact between the surface of the substrate/coating and the high-hardness Si_3_N_4_ ball at the onset of friction. Under the applied load, the ball embeds into the surface, causing a transition from point contact to area contact. This transition leads to a rapid increase in frictional force, resulting in a sharp rise in the friction coefficient. Notably, with increasing TiC content, the running-in period for the 30% TiC and 40% TiC coatings is further extended, with two distinct running-in phases observed: 0–200 s and 0–600 s, respectively. This can be attributed to the lower density of TiC. During the laser cladding process, unmolten TiC particles rise to the surface and form enrichment zones [38]. These TiC-rich regions increase the surface hardness of the coating, extending the time required for the transition from point to area contact and causing a more gradual increase in the friction coefficient. As wear progresses and these TiC-enriched regions are worn away, the friction coefficient rises again. Once the wear time reaches 600 s, all coatings and the substrate enter the stable wear stage. At this stage, the friction coefficient of the substrate is the highest, around 0.66, while the friction coefficients of the 10% TiC, 20% TiC, and 40% TiC coatings show little variation, remaining around 0.6. However, the friction coefficient of the 30% TiC coating is significantly lower than that of the 40% TiC coating, at approximately 0.55. This is primarily due to the excessive TiC content and the presence of cracks at the boundaries of unmolten TiC particles. During wear process, these unmolten TiC particles can detach from the coating, especially at crack sites where the bonding between the TiC and the coating matrix is weak. The detached TiC particles act as hard abrasives during friction, accelerating wear and causing an increase in the friction coefficient.

**Fig 11:**
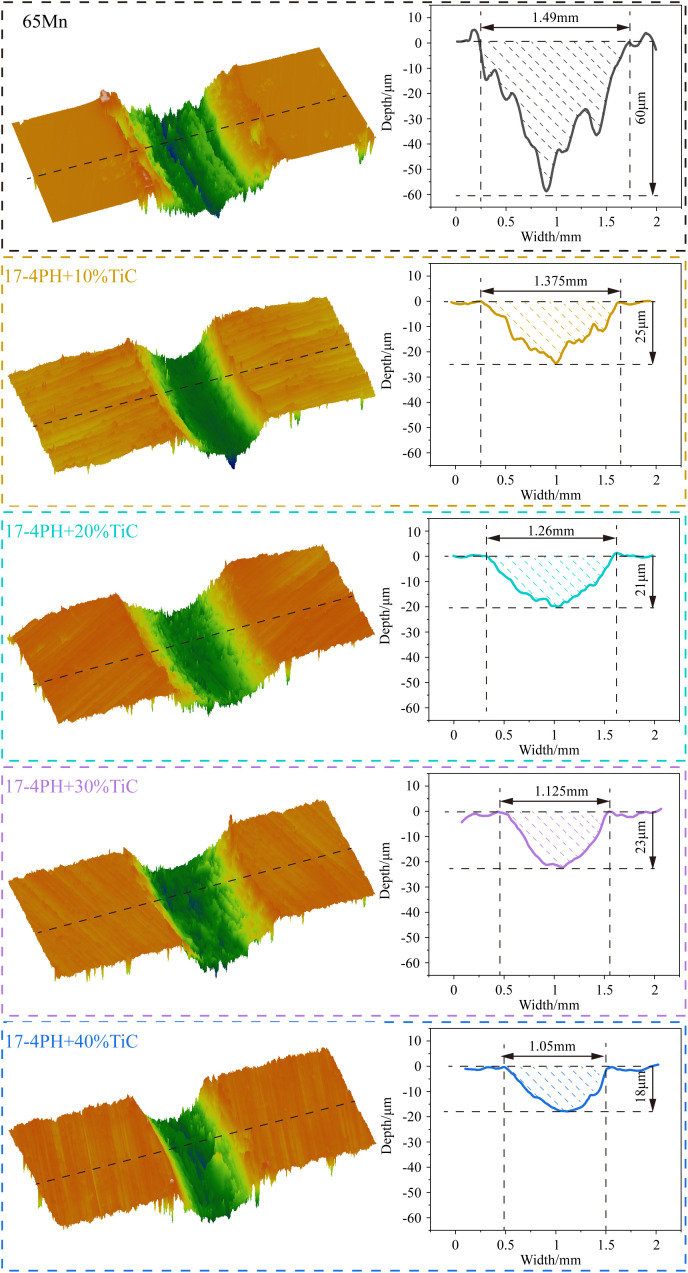
Abrasion morphology of 65Mn substrate and 17-4PH-xTiC coatings.

The wear morphology of TiC coatings with varying contents at room temperature is presented in Fig. 11, with the corresponding wear properties summarized in Table 1. The substrate 65Mn exhibits broader and deeper wear tracks compared with the coating. The presence of pronounced grooves at the base of the substrate’s abrasion marks suggests that abrasive wear is the predominant mechanism. As the TiC content in the coating increases, a discernible reduction in the width of the abrasion marks from 1.375 mm to 1.05 mm occurs, as well as a decrease in depth from 25 μm to 18 μm, indicating a significant enhancement in the coating’s abrasion resistance relative to the substrate. The wear rate of the coating diminishes by 44.1%–68.1% with increasing TiC content, which results in a marked increase in the coating’s wear resistance compared with that of the substrate. However, while the wear resistance of the coating improves, the addition of 40% TiC yields only a marginal enhancement over the 30% TiC coating, and the 40% TiC coating exhibits cracking. Consequently, the 17–4PH-30% TiC powder is selected as the optimal coating material for the blade.

**Table 1:** Wear quality of 17-4PH-xTiC coatings after friction wear tests.

weight	65Mn	10%TiC Coating	20%TiC Coating	30%TiC Coating	40%TiC Coating
Average weight loss (mg)	13.95	7.80	6.30	4.80	4.45

**Fig 12:**
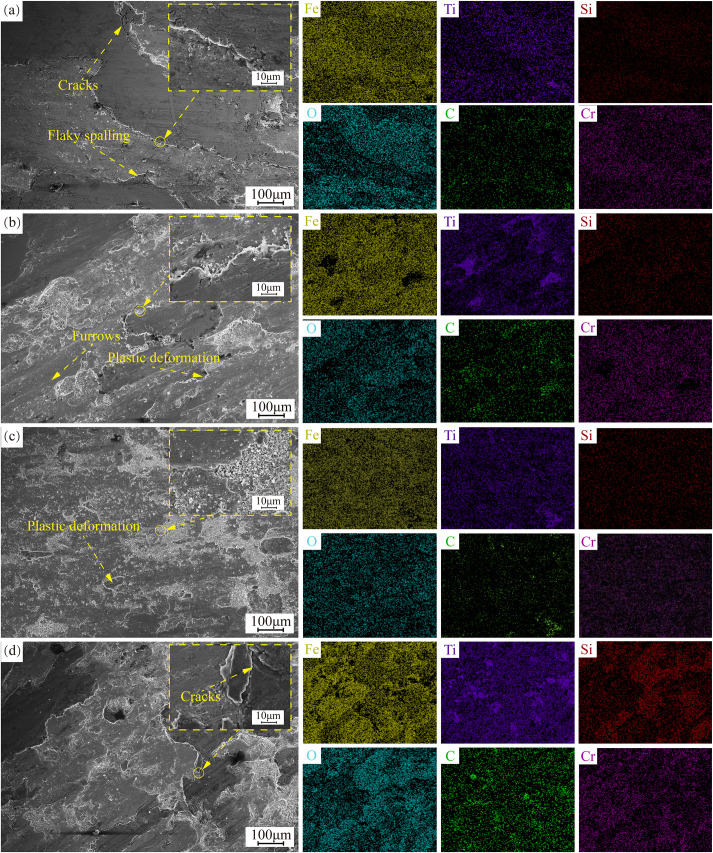
SEM morphology and EDS surface-scanning energy spectra of 17-4PH-xTiC coated abrasion marks: (a) 10%TiC; (b) 20%TiC; (c) 30%TiC; (d) 40%TiC.

Fig 12 presents magnified images of the worn surface regions and the corresponding elemental distributions for all samples, where signals of Fe, Ti, Cr, and O consistently appear on the friction surface. Notably, Si is detected at the bottom of the wear tracks, which is not a component of the coating. Moreover, the signal of Si intensifies with the increasing TiC content. In Fig 12(b), small furrows can also be observed, indicating the occurrence of abrasive wear.

In the early stage of wear, the coating surface first undergoes plastic deformation, followed by plastic fatigue wear. At this point, the 17−4PH layer in the coating detaches from the friction surface in the form of wear debris. During the friction process, the detached coating debris undergoes continuous deformation under the influence of pressure and shear forces [39]. The heat generated in this process causes the smaller debris to oxidize at high temperatures. Regions rich in oxygen coincide with regions rich in Ti, confirming the oxidation of Ti. These observations indicate that adhesive wear and oxidative wear occur at the bottom of the coating, which is further validated by the EDS results [40].As the surface wear progresses to expose the TiC particles, the TiC particles are revealed at the friction interface. With the relative motion of the friction pairs, the edges of the TiC particles are pushed towards the harder regions by the wear debris and become obstructed. However, under the continuous action of pressure and shear forces, the movement of the debris is inevitable [41]. As a result, instead of accumulating at the edges of the TiC particles, the debris covers the TiC particle surfaces, forming a mechanically mixed layer. EDS spectra show a significant presence of Ti on the worn surface, indicating that the exposed TiC particles collided with the dense Si_3_N_4_ spheres, causing the TiC particles to fracture. These fractured particles mixed with the wear debris detached from the iron substrate and underwent further fragmentation under pressure and shear forces. This process explains the persistent Ti signal across the entire friction surface. Moreover, during these collisions, the Si_3_N_4_ spheres also fractured. As the TiC content in the coating increased, the number of high-hardness TiC particles also rose, leading to more intense collisions between the Si_3_N_4_ spheres and TiC, which in turn increased the amount of fractured Si_3_N_4_ particles on the worn surface. This explains the source of Si in the EDS spectrum. The participation of these fine particles resulted in the formation of grooves on the friction surface. Therefore, both adhesive wear and abrasive wear occurred simultaneously during the friction process.

### Field test

**Fig 13:**
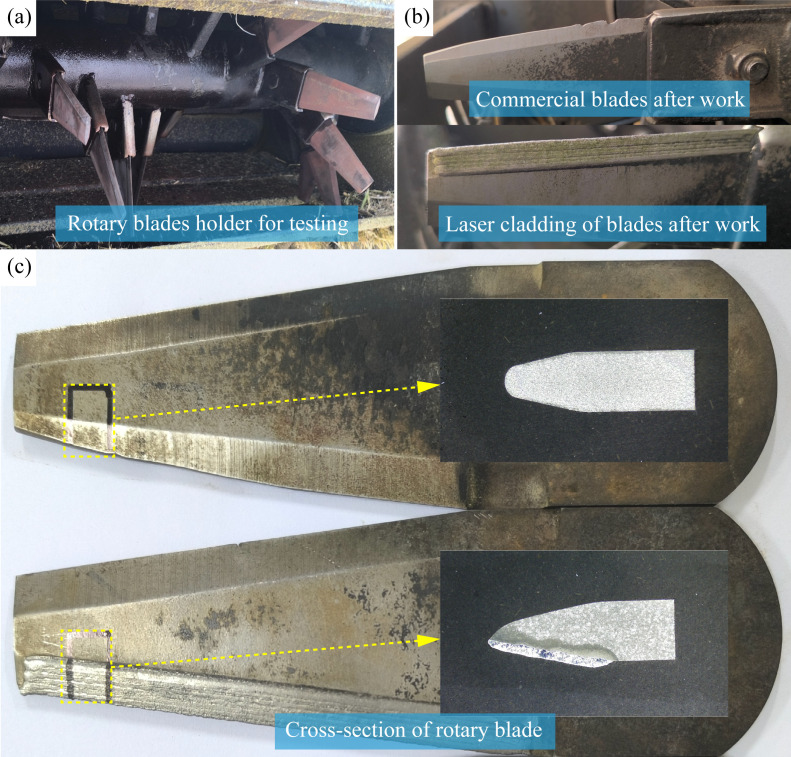
(a) The installation method of blade holder, (b) Blades in operation, (c) The blade after 120h of wheat harvesting.

During harvesting operations, combine harvester knives rotate at high speeds. The tip of the knife is farther from the rotation axis of the knife frame, resulting in a higher linear velocity at the tip than that in other positions. Consequently, the tip experiences greater impact force during crop harvesting, making it more susceptible to wear and failure. As the knife wears, the cutting angle increases, degrading the crushing effect on crop stalks and preventing crushed straw from meeting field return standards [42–45]. The wear condition of the blade tip can effectively reflect the service life of the blade. The installation method of the blade is shown in Fig 13(a), from which the blades are mounted on the rotating cutter head in a helical progression, ensuring consistent working conditions for each blade. Fig 13(b) compares the wear conditions of the blades after 80 h of operation. At this point, the tip of the commercially available blade shows severe wear, whereas the laser-clad blade exhibits significantly less wear.

Fig 13(c) shows the macroscopic surface morphology of the laser-clad blade and the commercially available blade after 120 h of wheat harvesting. After 120 h of operation, the wear of the commercially available blade is significantly greater than that of the laser-enhanced blade. The tip of the laser-clad blade shows almost no change after use, whereas the tip of the commercially available blade has been worn into a sloped surface. Examination of the cross section of the blades reveals that the wear on both sides of the blade of the commercially available blade is even and forms an arc shape. At this point, the commercially available blade has completely failed and can no longer meet the requirements for straw returning to the field.

For the laser-clad blade, the presence of the enhanced coating results in wear mainly occurring on the softer side of the blade during the wheat harvesting process. Owing to the uneven wear rate, a self-sharpening effect is formed at the cutting edge of the laser-clad blade [46]. The self-sharpening effect is achieved by the significant hardness difference between the cladded surface and the untreated blade body. The differential wear rates naturally regenerate a sharp cutting-edge during service. This capacity to regenerate a sharp edge effect allows the laser-clad blade to have a smaller blade angle compared with the commercially available blade after completing the wheat harvest, thereby improving the quality of straw chopping while extending the service life of the blade.

**Fig 14:**
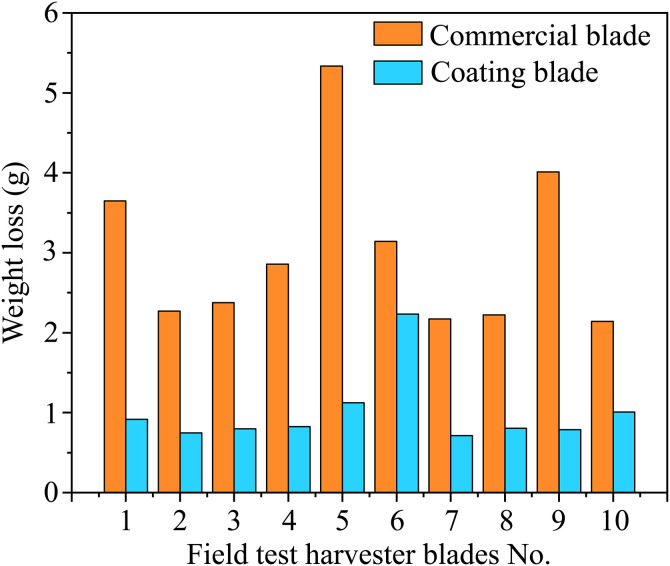
Comparison of wear quality of different blades after field tests.

The two blades are weighed after 120 h of harvesting, and the wear data are shown in Fig 14. The average wear of the laser-coated blade is 0.9950 g, whereas the average wear of the commercially available blade is 3.0173 g. Compared with that of the commercially available blade, the wear of the laser-coated blade is decreased by 67%. The edge of the commercially available blade has completely failed because of abrasion and cannot meet the requirements of wheat straw crushing, whereas the laser-coated blade can still continue to work. Based on the combine harvester crushing blade surface, laser cladding 17–4PH-30% TiC can effectively reduce blade wear and prolong the service life of the blade.

## Conclusion

In this study, 17–4PH-xTiC coatings were fabricated via laser cladding technology on the surface of 65Mn steel. The objective was to investigate the influence of TiC content on various properties of the coatings, including macro-morphology, microstructure, physical phase composition, hardness, and wear resistance. An optimal powder ratio was identified for creating wear-resistant coatings on the blade component of agricultural harvester blades, followed by field tests. The research and analysis led to the following conclusions:

1) Increasing TiC content promotes grain refinement through nucleation of decomposed TiC, enhancing coating properties. However, excessive TiC (>40%) results in unmelted particles and cracking due to thermal expansion mismatch.
2) Higher TiC content increases carbide hard phases, significantly improving hardness and wear resistance. The coatings exhibited 1.73–2.69 times higher hardness and 44.1%–68.1% lower wear loss than the substrate.
3) A blend with 30% TiC was applied to harvester blades. Field tests demonstrated superior wear resistance and a self-sharpening effect, reducing wear by 67% compared to commercial blades. The laser-clad blade remained functional even after conventional blade failure.
4) Laser cladding effectively extends blade service life and offers a viable approach for enhancing agricultural cutter performance. Future work will focus on optimizing process parameters to further improve blade durability.
